# 3,4,6-Tri­amino-*N*-phenyl­thieno[2,3-*b*]pyridine-2-carboxamide

**DOI:** 10.1107/S1600536814013981

**Published:** 2014-06-25

**Authors:** Shaaban K. Mohamed, Joel T. Mague, Mehmet Akkurt, Bahgat R. M. Hussein, Mustafa R. Albayati

**Affiliations:** a Chemistry and Environmental Division, Manchester Metropolitan University, Manchester M1 5GD, England; b Chemistry Department, Faculty of Science, Minia University, 61519 El-Minia, Egypt; c Department of Chemistry, Tulane University, New Orleans, LA 70118, USA; d Department of Physics, Faculty of Sciences, Erciyes University, 38039 Kayseri, Turkey; e Chemistry Department, Faculty of Science, Sohag University, 82524 Sohag, Egypt; f Kirkuk University, College of Science, Department of Chemistry, Kirkuk, Iraq

**Keywords:** crystal structure

## Abstract

In the title compound, C_14_H_13_N_5_OS, the dihedral angle between the fused ring system (r.m.s. deviation = 0.028 Å) and the phenyl ring is 48.24 (4)°. The mol­ecule features both an intra­molecular N—H⋯O and an N—H⋯N hydrogen bond. In the crystal, mol­ecules are linked by N—H⋯O and N—H⋯N hydrogen bonds, generating a three-dimensional network. A weak N—H⋯π inter­action is also observed.

## Related literature   

For background to thieno­pyridine-containing compounds, see: Boschelli *et al.* (2008 ▶); Bakhite *et al.* (2002 ▶); Schnute *et al.* (2007 ▶).
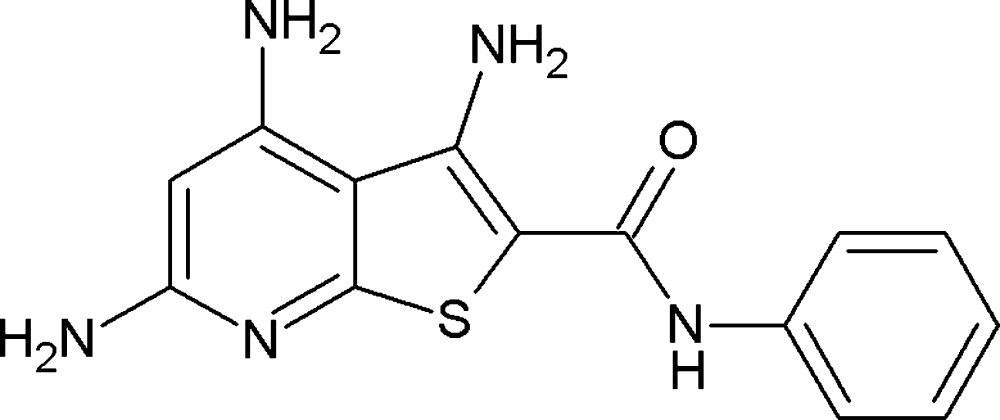


## Experimental   

### 

#### Crystal data  

C_14_H_13_N_5_OS
*M* *_r_* = 299.36
Monoclinic, 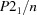

*a* = 5.2732 (7) Å
*b* = 21.028 (3) Å
*c* = 11.9777 (16) Å
β = 93.969 (2)°
*V* = 1325.0 (3) Å^3^
*Z* = 4
Mo *K*α radiation
μ = 0.25 mm^−1^
*T* = 150 K
0.21 × 0.13 × 0.09 mm

#### Data collection  

Bruker SMART APEX CCD diffractometer
Absorption correction: multi-scan (*SADABS*; Bruker, 2013 ▶) *T*_min_ = 0.85, *T*_max_ = 0.98
24103 measured reflections
3500 independent reflections
3017 reflections with *I* > 2σ(*I*)
*R*_int_ = 0.046

#### Refinement  

*R*[*F* ^2^ > 2σ(*F* ^2^)] = 0.037
*wR*(*F* ^2^) = 0.101
*S* = 1.06
3500 reflections
190 parameters
H-atom parameters constrained
Δρ_max_ = 0.42 e Å^−3^
Δρ_min_ = −0.25 e Å^−3^

### 

Data collection: *APEX2* (Bruker, 2013 ▶); cell refinement: *SAINT* (Bruker, 2013 ▶); data reduction: *SAINT*; program(s) used to solve structure: *SHELXT* (Bruker, 2013 ▶); program(s) used to refine structure: *SHELXL2014* (Sheldrick, 2008 ▶); molecular graphics: *DIAMOND* (Brandenburg & Putz, 2012 ▶); software used to prepare material for publication: *SHELXTL* (Sheldrick, 2008 ▶).

## Supplementary Material

Crystal structure: contains datablock(s) global, I. DOI: 10.1107/S1600536814013981/hb7235sup1.cif

Structure factors: contains datablock(s) I. DOI: 10.1107/S1600536814013981/hb7235Isup2.hkl

Supporting information file. DOI: 10.1107/S1600536814013981/hb7235Isup3.cml

CCDC reference: 1008299

Additional supporting information:  crystallographic information; 3D view; checkCIF report

## Figures and Tables

**Table 1:** Hydrogen-bond geometry (Å, °) *Cg*3 is the centroid of the C9–C14 phenyl ring.

*D*—H⋯*A*	*D*—H	H⋯*A*	*D*⋯*A*	*D*—H⋯*A*
N2—H2*A*⋯N4^i^	0.91	2.52	3.2226 (17)	134
N3—H3*A*⋯N1^ii^	0.91	2.07	2.9398 (17)	161
N3—H3*B*⋯N4	0.91	2.38	2.9802 (17)	124
N3—H3*B*⋯N2^iii^	0.91	2.41	3.2034 (16)	146
N4—H4*B*⋯O1	0.91	2.15	2.8387 (16)	132
N4—H4*B*⋯O1^iv^	0.91	2.32	2.9991 (16)	132
N2—H2*B*⋯*Cg*3^v^	0.91	2.56	3.4662 (14)	173

Symmetry codes: (i) 
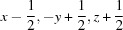
; (ii) 
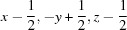
; (iii) 
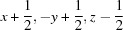
; (iv) 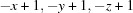; (v) 
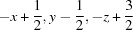
.

**Fig. 1.**
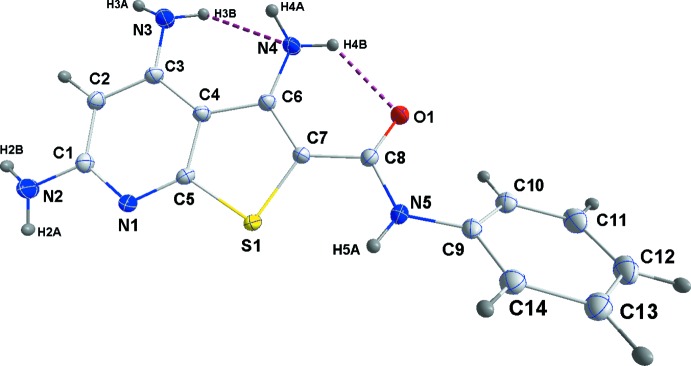
The title compound with 50% probability ellipsoids and with intramolecular hydrogen bonds shown as dotted lines.

**Fig. 2.**
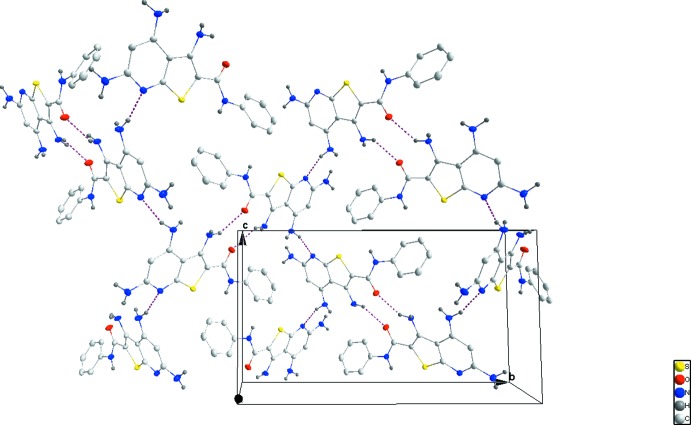
Packing viewed down the a axis showing a portion of the zigzag sheet with the intermolecular hydrogen bonds depicted by dotted lines.

**Fig. 3.**
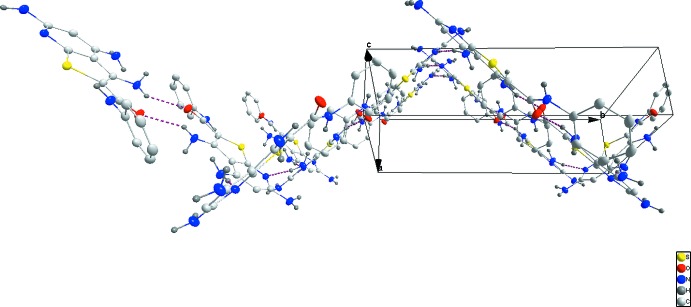
Packing viewed parallel to (101).

## supplementary crystallographic information

### S1. Comment

Thienopyridines and their analogs are an interesting class of molecules due to their extensive spectrum of pharmacological properties, for example anti-inflammatory (Boschelli *et al.*, 2008), anti-microbial (Bakhite *et al.*, 2002) and anti-viral (Schnute *et al.*, 2007) activities. As part of our program in the development of new heterocyclic molecules with potential bioactivities, we report in this study the synthesis and crystal structure determination of the title compound.

In the title compound, the fused ring system is nearly planar with an r.m.s. deviation of 0.028 Å and makes a dihedral angle of 48.24 (4)° with the terminal phenyl group. The conformation of the carboxamide group is partially determined by the intramolecular N4—H4B···O1 hydrogen bond (Fig. 1 and Table 1). In the solid, the molecules associate through pairwise intermolecular N4—H4B···O1 and single N3—H3a···N1 hydrogen bonds to form a three-dimensional network (Figs. 2 and 3 and Table 1). A weak N—H···π interaction is observed between the NH_2_ group (N2—H2B) and the centroid of the C9–C14 phenyl ring.

### S2. Experimental

A mixture of 2.7 mmol (500 mg) of 4,6-diamino-2-mercaptonicotinonitrile, 2.7 mmol (150 mg) potassium hydroxide and 1.86 mmol (320 mg) in 30 ml e thanol was stirred and refluxed for 3 h. The excess solvent was evaporated under vacuum and the resulting solid product was filtered off, washed with cold ethanol and recrystallized from ethanol to furnish colourless crystals (730 mg; 87% yield). Mp. 531 – 533 K.

IR (ν_max_, cm^-1^): 3462, 3402, 3352, (3NH_2_), 3213 (NH), 1645 (C=O), 1614 (C=N); ^1^HNMR (DMSO-d_6_), δ, p.p.m.: 8.88 (s, 1H, NH exchanged by D_2_O), 7.65–7.63 (d, J = 8 Hz, 2H, arom), 7.29–7.25 (t, J = 8 Hz, 2H, arom), 7.02–6.99 (m,, 3H, arom+ NH_2_ exchanged by D_2_O), 6.11 (s, 2H, NH_2_ exchanged by D_2_O), 6.02 (s, 2H, NH_2_ exchanged by D_2_O), 5.59 (s, 1H, CH pyridyl).

### S3. Refinement

H-atoms attached to carbon were placed in calculated positions (C—H = 0.95 Å) while those attached to nitrogen were placed in locations derived from a difference map and their coordinates adjusted to give N—H = 0.91 Å following an initial round of refinement to check the validity of the peak assignments. All were included as riding contributions with isotropic displacement parameters 1.2 times those of the attached atoms.

### Crystal data

**Table:**

C_14_H_13_N_5_OS	*F*(000) = 624
*M**_r_* = 299.36	*D*_x_ = 1.501 Mg m^−^^3^
Monoclinic, *P*2_1_/*n*	Mo *K*α radiation, λ = 0.71073 Å
Hall symbol: -P 2yn	Cell parameters from 9996 reflections
*a* = 5.2732 (7) Å	θ = 2.6–29.1°
*b* = 21.028 (3) Å	µ = 0.25 mm^−^^1^
*c* = 11.9777 (16) Å	*T* = 150 K
β = 93.969 (2)°	Block, colourless
*V* = 1325.0 (3) Å^3^	0.21 × 0.13 × 0.09 mm
*Z* = 4	

### Data collection

**Table:**

Bruker SMART APEX CCD diffractometer	3500 independent reflections
Radiation source: fine-focus sealed tube	3017 reflections with *I* > 2σ(*I*)
Graphite monochromator	*R*_int_ = 0.046
Detector resolution: 8.3660 pixels mm^-1^	θ_max_ = 29.1°, θ_min_ = 1.9°
φ and ω scans	*h* = −6→7
Absorption correction: multi-scan (*SADABS*; Bruker, 2013)	*k* = −28→28
*T*_min_ = 0.85, *T*_max_ = 0.98	*l* = −16→16
24103 measured reflections	

### Refinement

**Table:**

Refinement on *F*^2^	0 restraints
Least-squares matrix: full	Hydrogen site location: mixed
*R*[*F*^2^ > 2σ(*F*^2^)] = 0.037	H-atom parameters constrained
*wR*(*F*^2^) = 0.101	*w* = 1/[σ^2^(*F*_o_^2^) + (0.0512*P*)^2^ + 0.5972*P*] where *P* = (*F*_o_^2^ + 2*F*_c_^2^)/3
*S* = 1.06	(Δ/σ)_max_ = 0.001
3500 reflections	Δρ_max_ = 0.42 e Å^−^^3^
190 parameters	Δρ_min_ = −0.25 e Å^−^^3^

### Special details

**Table:**

Geometry. Bond distances, angles *etc*. have been calculated using the rounded fractional coordinates. All su's are estimated from the variances of the (full) variance-covariance matrix. The cell e.s.d.'s are taken into account in the estimation of distances, angles and torsion angles
Refinement. Refinement on *F*^2^ for ALL reflections except those flagged by the user for potential systematic errors. Weighted *R*-factors *wR* and all goodnesses of fit *S* are based on *F*^2^, conventional *R*-factors *R* are based on *F*, with *F* set to zero for negative *F*^2^. The observed criterion of *F*^2^ > σ(*F*^2^) is used only for calculating -*R*-factor-obs *etc*. and is not relevant to the choice of reflections for refinement. *R*-factors based on *F*^2^ are statistically about twice as large as those based on *F*, and *R*-factors based on ALL data will be even larger.

### Fractional atomic coordinates and isotropic or equivalent isotropic displacement parameters (Å^2^)

**Table:**

	*x*	*y*	*z*	*U*_iso_*/*U*_eq_	
S1	0.40493 (6)	0.35471 (2)	0.83539 (3)	0.0168 (1)	
O1	0.6615 (2)	0.47538 (5)	0.61593 (8)	0.0249 (3)	
N1	0.0339 (2)	0.26672 (5)	0.82888 (9)	0.0169 (3)	
N2	−0.2636 (2)	0.18599 (6)	0.82055 (10)	0.0230 (3)	
N3	−0.1968 (2)	0.31627 (6)	0.49527 (10)	0.0220 (3)	
N4	0.2168 (2)	0.41258 (6)	0.52579 (9)	0.0209 (3)	
N5	0.7313 (2)	0.47187 (6)	0.80636 (10)	0.0196 (3)	
C1	−0.1541 (3)	0.23617 (6)	0.76882 (11)	0.0168 (3)	
C2	−0.2385 (3)	0.25273 (6)	0.65898 (11)	0.0170 (3)	
C3	−0.1212 (3)	0.30131 (6)	0.60362 (11)	0.0161 (3)	
C4	0.0807 (2)	0.33451 (6)	0.66470 (10)	0.0150 (3)	
C5	0.1430 (2)	0.31391 (6)	0.77427 (10)	0.0149 (3)	
C6	0.2455 (3)	0.38441 (6)	0.63091 (11)	0.0157 (3)	
C7	0.4308 (3)	0.40001 (6)	0.71359 (10)	0.0163 (3)	
C8	0.6148 (3)	0.45111 (6)	0.70611 (11)	0.0172 (3)	
C9	0.9058 (3)	0.52266 (6)	0.82104 (11)	0.0178 (3)	
C10	1.0866 (3)	0.53587 (7)	0.74444 (12)	0.0199 (4)	
C11	1.2629 (3)	0.58416 (7)	0.76735 (14)	0.0245 (4)	
C12	1.2616 (3)	0.61978 (7)	0.86512 (14)	0.0261 (4)	
C13	1.0791 (3)	0.60708 (7)	0.94063 (13)	0.0236 (4)	
C14	0.9013 (3)	0.55895 (7)	0.91876 (12)	0.0207 (4)	
H2	−0.37710	0.23050	0.62220	0.0200*	
H2A	−0.17740	0.16940	0.88230	0.0280*	
H2B	−0.36030	0.15820	0.77790	0.0280*	
H3A	−0.29050	0.28560	0.45810	0.0260*	
H3B	−0.07270	0.33330	0.45530	0.0260*	
H4A	0.05280	0.42370	0.50670	0.0250*	
H4B	0.33410	0.44320	0.51430	0.0250*	
H5A	0.66340	0.45840	0.87010	0.0230*	
H10	1.08930	0.51200	0.67710	0.0240*	
H11	1.38620	0.59300	0.71530	0.0290*	
H12	1.38390	0.65240	0.88020	0.0310*	
H13	1.07580	0.63140	1.00740	0.0280*	
H14	0.77650	0.55070	0.97040	0.0250*	

### Atomic displacement parameters (Å^2^)

**Table:**

	*U*^11^	*U*^22^	*U*^33^	*U*^12^	*U*^13^	*U*^23^
S1	0.0174 (2)	0.0186 (2)	0.0138 (2)	−0.0032 (1)	−0.0034 (1)	0.0018 (1)
O1	0.0273 (6)	0.0270 (5)	0.0196 (5)	−0.0099 (4)	−0.0033 (4)	0.0059 (4)
N1	0.0165 (6)	0.0174 (5)	0.0167 (5)	−0.0011 (4)	0.0000 (4)	0.0018 (4)
N2	0.0234 (6)	0.0229 (6)	0.0223 (6)	−0.0066 (5)	−0.0008 (5)	0.0051 (5)
N3	0.0243 (6)	0.0263 (6)	0.0147 (5)	−0.0084 (5)	−0.0037 (4)	0.0006 (5)
N4	0.0256 (6)	0.0215 (6)	0.0151 (5)	−0.0059 (5)	−0.0027 (5)	0.0044 (4)
N5	0.0211 (6)	0.0193 (6)	0.0180 (5)	−0.0064 (4)	−0.0012 (4)	0.0003 (4)
C1	0.0152 (6)	0.0166 (6)	0.0187 (6)	0.0005 (5)	0.0028 (5)	−0.0001 (5)
C2	0.0155 (6)	0.0177 (6)	0.0175 (6)	−0.0021 (5)	−0.0001 (5)	−0.0016 (5)
C3	0.0162 (6)	0.0173 (6)	0.0145 (6)	0.0001 (5)	−0.0003 (5)	−0.0015 (5)
C4	0.0168 (6)	0.0149 (6)	0.0131 (6)	−0.0007 (5)	−0.0006 (5)	−0.0002 (4)
C5	0.0141 (6)	0.0156 (6)	0.0145 (6)	−0.0001 (4)	−0.0016 (5)	−0.0011 (4)
C6	0.0178 (6)	0.0151 (6)	0.0140 (5)	−0.0009 (5)	0.0003 (5)	−0.0005 (4)
C7	0.0181 (6)	0.0163 (6)	0.0142 (6)	−0.0020 (5)	−0.0004 (5)	0.0019 (5)
C8	0.0160 (6)	0.0165 (6)	0.0188 (6)	−0.0012 (5)	−0.0018 (5)	0.0004 (5)
C9	0.0162 (6)	0.0147 (6)	0.0218 (6)	−0.0004 (5)	−0.0044 (5)	0.0019 (5)
C10	0.0169 (6)	0.0184 (6)	0.0242 (7)	0.0010 (5)	−0.0008 (5)	0.0001 (5)
C11	0.0158 (7)	0.0237 (7)	0.0338 (8)	−0.0020 (5)	−0.0003 (6)	0.0045 (6)
C12	0.0206 (7)	0.0187 (7)	0.0375 (8)	−0.0043 (5)	−0.0079 (6)	0.0019 (6)
C13	0.0246 (7)	0.0184 (7)	0.0264 (7)	−0.0003 (5)	−0.0081 (6)	−0.0020 (5)
C14	0.0201 (7)	0.0200 (6)	0.0214 (6)	−0.0009 (5)	−0.0030 (5)	0.0000 (5)

### Geometric parameters (Å, º)

**Table:**

S1—C5	1.7435 (12)	C2—C3	1.3866 (19)
S1—C7	1.7555 (13)	C3—C4	1.4312 (19)
O1—C8	1.2348 (17)	C4—C6	1.4383 (18)
N1—C1	1.3470 (18)	C4—C5	1.3994 (17)
N1—C5	1.3400 (16)	C6—C7	1.382 (2)
N2—C1	1.3712 (18)	C7—C8	1.455 (2)
N3—C3	1.3677 (18)	C9—C10	1.396 (2)
N4—C6	1.3902 (17)	C9—C14	1.399 (2)
N5—C8	1.3812 (18)	C10—C11	1.391 (2)
N5—C9	1.4128 (18)	C11—C12	1.391 (2)
N2—H2B	0.9100	C12—C13	1.391 (2)
N2—H2A	0.9100	C13—C14	1.392 (2)
N3—H3B	0.9100	C2—H2	0.9500
N3—H3A	0.9100	C10—H10	0.9500
N4—H4A	0.9100	C11—H11	0.9500
N4—H4B	0.9100	C12—H12	0.9500
N5—H5A	0.9100	C13—H13	0.9500
C1—C2	1.4033 (19)	C14—H14	0.9500
			
C5—S1—C7	91.32 (6)	N4—C6—C7	125.12 (13)
C1—N1—C5	114.68 (11)	C4—C6—C7	112.50 (11)
C8—N5—C9	126.43 (12)	C6—C7—C8	124.95 (12)
C1—N2—H2B	119.00	S1—C7—C6	112.03 (10)
H2A—N2—H2B	116.00	S1—C7—C8	122.86 (10)
C1—N2—H2A	117.00	O1—C8—N5	121.75 (13)
C3—N3—H3B	115.00	O1—C8—C7	122.24 (12)
H3A—N3—H3B	114.00	N5—C8—C7	116.00 (11)
C3—N3—H3A	114.00	C10—C9—C14	119.56 (13)
C6—N4—H4B	114.00	N5—C9—C10	122.49 (12)
H4A—N4—H4B	115.00	N5—C9—C14	117.90 (13)
C6—N4—H4A	112.00	C9—C10—C11	119.54 (13)
C9—N5—H5A	115.00	C10—C11—C12	121.14 (15)
C8—N5—H5A	117.00	C11—C12—C13	119.21 (14)
N1—C1—C2	123.80 (12)	C12—C13—C14	120.29 (14)
N2—C1—C2	119.90 (13)	C9—C14—C13	120.26 (14)
N1—C1—N2	116.30 (12)	C1—C2—H2	120.00
C1—C2—C3	120.54 (13)	C3—C2—H2	120.00
N3—C3—C4	122.04 (12)	C9—C10—H10	120.00
N3—C3—C2	120.86 (13)	C11—C10—H10	120.00
C2—C3—C4	117.10 (12)	C10—C11—H11	119.00
C5—C4—C6	112.49 (11)	C12—C11—H11	119.00
C3—C4—C6	130.89 (12)	C11—C12—H12	120.00
C3—C4—C5	116.52 (11)	C13—C12—H12	120.00
S1—C5—C4	111.67 (9)	C12—C13—H13	120.00
N1—C5—C4	127.31 (11)	C14—C13—H13	120.00
S1—C5—N1	120.92 (9)	C9—C14—H14	120.00
N4—C6—C4	122.39 (12)	C13—C14—H14	120.00
			
C7—S1—C5—N1	176.86 (11)	C6—C4—C5—S1	−0.75 (14)
C7—S1—C5—C4	0.24 (10)	C6—C4—C5—N1	−177.10 (12)
C5—S1—C7—C6	0.35 (11)	C3—C4—C6—N4	4.6 (2)
C5—S1—C7—C8	175.81 (12)	C3—C4—C6—C7	−175.08 (13)
C5—N1—C1—N2	177.57 (11)	C5—C4—C6—N4	−179.26 (12)
C5—N1—C1—C2	−1.99 (19)	C5—C4—C6—C7	1.02 (16)
C1—N1—C5—S1	−175.21 (10)	N4—C6—C7—S1	179.46 (11)
C1—N1—C5—C4	0.84 (18)	N4—C6—C7—C8	4.1 (2)
C9—N5—C8—O1	2.2 (2)	C4—C6—C7—S1	−0.83 (15)
C9—N5—C8—C7	−176.62 (13)	C4—C6—C7—C8	−176.18 (13)
C8—N5—C9—C10	−36.6 (2)	S1—C7—C8—O1	167.43 (11)
C8—N5—C9—C14	146.03 (14)	S1—C7—C8—N5	−13.78 (18)
N1—C1—C2—C3	2.8 (2)	C6—C7—C8—O1	−17.7 (2)
N2—C1—C2—C3	−176.78 (13)	C6—C7—C8—N5	161.09 (14)
C1—C2—C3—N3	177.76 (13)	N5—C9—C10—C11	−176.08 (14)
C1—C2—C3—C4	−2.1 (2)	C14—C9—C10—C11	1.2 (2)
N3—C3—C4—C5	−178.88 (12)	N5—C9—C14—C13	176.12 (13)
N3—C3—C4—C6	−2.9 (2)	C10—C9—C14—C13	−1.3 (2)
C2—C3—C4—C5	1.00 (18)	C9—C10—C11—C12	−0.3 (2)
C2—C3—C4—C6	176.98 (14)	C10—C11—C12—C13	−0.6 (2)
C3—C4—C5—S1	175.96 (9)	C11—C12—C13—C14	0.5 (2)
C3—C4—C5—N1	−0.39 (19)	C12—C13—C14—C9	0.5 (2)

### Hydrogen-bond geometry (Å, º)

Cg3 is the centroid of the C9–C14 phenyl ring.

**Table:**

*D*—H···*A*	*D*—H	H···*A*	*D*···*A*	*D*—H···*A*
N2—H2*A*···N4^i^	0.91	2.52	3.2226 (17)	134
N3—H3*A*···N1^ii^	0.91	2.07	2.9398 (17)	161
N3—H3*B*···N4	0.91	2.38	2.9802 (17)	124
N3—H3*B*···N2^iii^	0.91	2.41	3.2034 (16)	146
N4—H4*B*···O1	0.91	2.15	2.8387 (16)	132
N4—H4*B*···O1^iv^	0.91	2.32	2.9991 (16)	132
N2—H2*B*···*Cg*3^v^	0.91	2.56	3.4662 (14)	173

Symmetry codes: (i) *x*−1/2, −*y*+1/2, *z*+1/2; (ii) *x*−1/2, −*y*+1/2, *z*−1/2; (iii) *x*+1/2, −*y*+1/2, *z*−1/2; (iv) −*x*+1, −*y*+1, −*z*+1; (v) −*x*+1/2, *y*−1/2, −*z*+3/2.

## References

[bb1] Bakhite, E. A., Abdel-Rahman, A. E., Mohamed, O. S. & Thabet, E. A. (2002). *Bull. Korean Chem. Soc.* **23**, 1709–1714.

[bb2] Boschelli, D. H., Wu, B., Barrios, S. A. C., Chen, J., Asselin, M., Cole, D. C., Lee, J., Yang, X. & Chaudhary, D. (2008). *Bioorg. Med. Chem. Lett.* **18**, 2850–2853. 10.1016/j.bmcl.2008.03.077 18434148

[bb3] Brandenburg, K. & Putz, H. (2012). *DIAMOND* Crystal Impact GbR, Bonn, Germany.

[bb4] Bruker (2013). *APEX2*, *SADABS* and *SAINT* Bruker AXS Inc., Madison, Wisconsin, USA.

[bb5] Schnute, M. E., Anderson, D. J., Brideau, R. J., Ciske, F. L. & Collier, S. A. (2007). *Bioorg. Med. Chem. Lett.* **17**, 3349–3353. 10.1016/j.bmcl.2007.03.102 17434304

[bb6] Sheldrick, G. M. (2008). *Acta Cryst.* A**64**, 112–122. 10.1107/S0108767307043930 18156677

